# Study on the Electrospinning of Gelatin/Pullulan Composite Nanofibers

**DOI:** 10.3390/polym11091424

**Published:** 2019-08-30

**Authors:** Yuanduo Wang, Ziyang Guo, Yongfang Qian, Zhen Zhang, Lihua Lyu, Ying Wang, Fang Ye

**Affiliations:** 1 School of Textile and Material Engineering, Dalian Polytechnic University, Dalian 116034, China; 2 Sinomatech Membrane Material Company, Nanjing 211112, China

**Keywords:** electrospinning, gelatin, pullulan, nanofiber, tissue engineering scaffold

## Abstract

In this study, gelatin and pullulan were successfully prepared as a novel type of protein–polysaccharide composite nanofibrous membrane by electrospinning at room temperature with deionized water as the solvent. The effects of gelatin content on the properties of the solution, as well as the morphology of the resultant nanofibers, were investigated. Scanning electron microscopy (SEM) was utilized to observe the surface morphology. Fourier transform infrared spectroscopy (FTIR) was used to study the interaction between gelatin and pullulan. Incorporation of pullulan with gelatin will improve the spinnability of the mixed aqueous solution due to lower surface tension. Moreover, the conductivity of the solution had a greater effect on the fiber diameters, and the as-spun fibers became thinner as the viscosity and the surface tension increased due to the addition of the polyelectrolyte gelatin. Gelatin and pullulan formed hydrogen bonds, and the intermolecular hydrogen bonds increased while the intramolecular hydrogen bond decreased, which resulted in better mechanical properties. The electrospun gelatin/pullulan nanofibers could mimic both the structure and the composition of the extracellular matrix, and thus could be applied in tissue engineering.

## 1. Introduction

Over decades, researchers have developed various methods to produce nanofibers. Electrospinning is a current technique that can continuously and effectively prepare nanofibers [1]. Doshi and Reneker reported that the basic principle of electrospinning is that the polymer droplet at the tip of the nozzle is deformed from a hemispherical shape to a conical shape, which is known as a Taylor cone, under an electrical force. The jet is ejected from the nozzle when the applied voltage is increased sufficiently to overcome the surface tension, and then begins to whip after a few centimeters. Finally, the nanofibers are deposited on the grounded collector with the evaporation of solvent or the solidification of the polymer melt [2,3]. The effect of parameters on the spinnability and morphology includes the properties of the solution and processing variables, as well as the environmental temperature and humidity [4]. By adjusting the polymer solution and manufacturing parameters, fibers with diameters ranging from several micrometers to several tens of nanometers can be obtained.

Recently, electrospinning of natural biopolymers has become a hot issue due to their excellent biocompatibility, high porosity, and better suitability for the human body in comparison to synthetic polymers [5,6]. Gelatin is a natural biopolymer, which is obtained by hydrolyzing the collagen of skin, tendons, cartilage, and bone [7]. Gelatin, with its good biocompatibility and biodegradation, attributed to its composition of 14% hydroxyproline, 16% proline, and 26% glycine [8], has been widely applied in many biomedical applications such as wound dressings and tissue engineering [9,10]. Gelatin has been incorporated with polysaccharides [11], chitosan [12], silk fibroin [13], and polycaprolactone (PCL) [14] to be electrospun, and the resulting morphologies, properties, and applications have been studied. Raut et al. demonstrated that the addition of gelatin could improve the hydrophilicity, biocompatibility, and blood compatibility of the polyurethane surface [15]. Li et al. found that gelatin added to hyaluronic acid (HA) solution can significantly improve the processing properties of HA solutions with high viscosity, and had broad application prospects in the biomedical field [11]. Fanaee et al. found that the combination of PCL with gelatin could improve the poor hydrophilicity, and thus be beneficial to increasing cell adhesion, migration, proliferation, and differentiation [16].

Pullulan is a microbial polysaccharide produced by a yeast-like fungus, characterized by the coexistence of α-(1→4) and α-(1→6) glycosidic bonds [17]. The linear chemical structure gives pullulan excellent solubility and spinnability. Commercially available, pullulan is a tasteless, white-colored powder that is highly water-soluble and nontoxic [18]. Due to its excellent properties, pullulan has been used in cosmetics, food, medicines, and biomedical applications [19,20,21]. Pullulan has been incorporated with PVA and montmorillonite to improve the mechanical properties and thermal performance of electrospun mats [22,23]. Fanaee’s results showed that the increased amount of pullulan in a blend increased the maximum stress and strain at rupture [24]. 

Extracellular matrix (ECM) is a complex composed of nanosized proteins and glycosaminoglycans (GAGs) [25]. Gelatin is a protein that is derived from the partial hydrolysis of collagens, while pullulan is a polysaccharide that has a structure similar to GAGs in the ECM [26]. Therefore, an electrospun gelatin and pullulan complex could mimic both the structure and composition of natural ECM. Polymer blending is an effective method to modify and improve the physical and chemical properties of polymer materials [27]. To the best of our knowledge, the electrospinning of gelatin and pullulan composites has been rarely reported. In this study, the effects of gelatin content on the properties of the solution, as well as the further effects on the as-spun nanofibers, were investigated.

## 2. Materials and Methods 

### 2.1. Materials

Gelatin (Type A, 300 bloom) was obtained from Sigma-Aldrich (St. Louis, MO, USA), and pullulan (food-grade) was purchased from Hayashibara Biochemical Laboratories Inc. (Okayama, Japan). Deionized water (D.I. water) was used to prepare all aqueous solutions.

### 2.2. Solution Preparation

Gelatin and pullulan were dissolved in deionized water and stirred by a magnetic stirrer (RT10, IKA, German) at 30 °C for a minimum of 24 h. The concentration of the solution varied from 20% to 25% (*w*/*v*_H___2_O_), while the weight ratios of gelatin and pullulan were 25/75, 33/67, 40/60, and 50/50.

### 2.3. Characterization of the Solution Properties

#### 2.3.1. Viscosity Measurement

The viscosity of the blended solutions was determined by a digital viscosimeter (DV-79, Nirun, Shanghai, China) at room temperature over a measuring time of 2 min.

#### 2.3.2. Surface Tension Measurement

The surface tension of the solution was measured by a tensiometer (K100, KRUSS, Hamburg, Germany) using the flat plate method at room temperature after 30 min of preheating. Each sample was tested 5 times and averaged.

#### 2.3.3. Conductivity Measurement

The conductivity of blended solutions with different mass ratios was measured by a conductivity meter (DDS-307, Yidia, Shanghai, China) after 20 min of preheating. All measurements were carried out at room temperature. 

### 2.4. Electrospinning

The electrospinning from the mixed solutions was carried out at room temperature. The experimental setup used for electrospinning consisted of a syringe pump (789100C, Cole-Parmer, Chicago, IL, USA), on which a 2 mL syringe was connected to the stainless steel needle; a high-voltage power supply (JDF-1, Beijing, China) which generated DC voltage in a range of 0–50 kV; and a grounded plate receiver covered with aluminum foil. The pumping speed was set at 0.5 mL/h, and the applied voltage was set at 20 kV. The plate was placed 20 cm from the tip of the nozzle and used to collect the as-spun nanofibers.

### 2.5. Characterization of the Blended Gelatin/Pullulan Nanofibrous Membrane

#### 2.5.1. Morphological Characterization of Nanofiber Membrane

Aluminum foil with nanofiber membrane was sprayed with platinum. The morphology of the electrospun nanofibers was observed with a scanning electron microscope (JSM-7800F, JEOL Ltd., Tokyo, Japan) under high vacuum. The applied accelerating voltage was 15 kV, and the working distance was 10 mm. The diameters of fibers were measured randomly on 60 fibers per sample using the Nano Measurer 1.2 software (Surface Chemistry and Catalysis Laborator, Shanghai, China), and they provided the average diameter as well as the size distribution of the fibers.

#### 2.5.2. Fourier Transform Infrared Spectroscopy (FTIR) Analysis

Gelatin, pullulan, and their composite membranes were analyzed by Fourier transform infrared spectroscopy (FTIR, iS50, Thermo Science, Waltham, MA, USA, USA) in a scanning range of 500–4000 cm^−1^ for 64 scans at a spectral resolution of 4 cm^−1^.

#### 2.5.3. Tensile Strength Measurement

The mechanical properties of the tensile stress and strain were performed using electronic single yarn strength testing machines (LLY06E, Laizhou, China). The samples were 50 mm in length and 10 mm in width. The gauge length between the two holders was 30 mm, and the cross-head speed was 10 mm/min. The test was carried out under constant temperature and humidity condition of 25 °C and 65% humidity. 

### 2.6. Statistical Analysis

All measurements in this work were conducted at least 5 times, and then the average value was obtained through statistical analysis. 

## 3. Results and Discussion

### 3.1. Characterization of the Solution Properties

#### 3.1.1. Viscosity of the Solution

**Figure 1:**
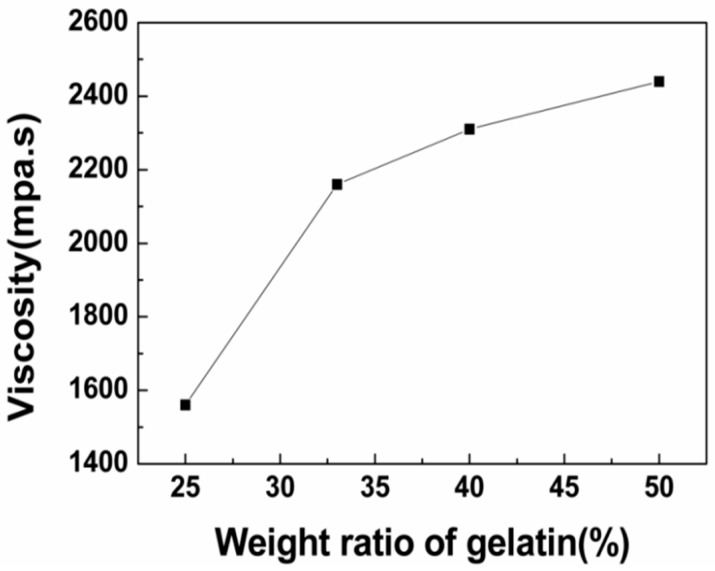
The relationship between the viscosity of the spinning solution and the weight ratios of gelatin (total polymer concentration = 25% *w*/*v*).

Fiber morphology was greatly affected by the viscosity of the solution. Change in the polymer concentration will vary the viscosity of the solution. Therefore, the relationship between the viscosity of the solution and gelatin content in the dope was studied. The viscosity of the solution varied with the gelatin content, as shown in Figure 1. The viscosity of the solution increased with the increasing gelatin content. The reason for this could be that the internal friction resistance of the spinning solution increased and the molecular fluidity decreased [28].

#### 3.1.2. Surface Tension Analysis of Blended Gelatin/Pullulan Solutions

**Figure 2:**
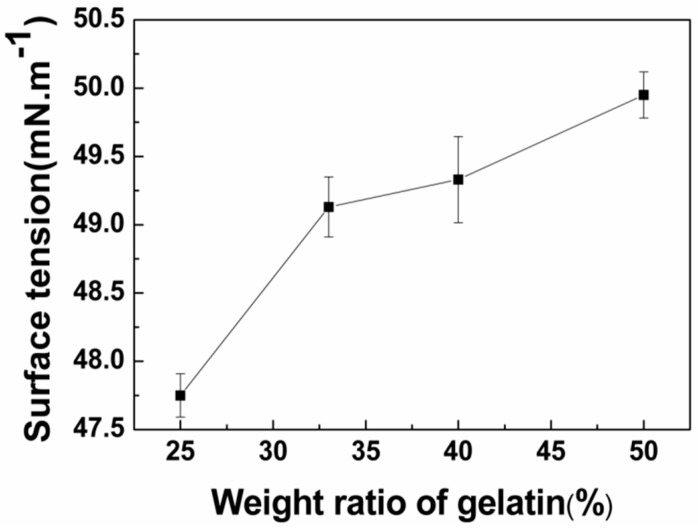
The relationship between the surface tension of blended gelatin/pullulan solutions and the weight ratios of gelatin (total polymer concentration = 25% *w*/*v*).

The reduction of surface tension is beneficial to the preparation of bead-free fibers [29]. The surface tension of the gelatin solutions was influenced by the concentration, temperature, and other factors [30]. The surface tension varied with gelatin content at the same concentration, as shown in Figure 2. The surface tensions of blended gelatin/pullulan solutions increased with increasing gelatin content. However, the surface tensions of all of the solutions were less than the surface tension of pure water (70 mm·m^−1^).

#### 3.1.3. Conductivity Analysis of Blended Gelatin/Pullulan Solutions

**Figure 3:**
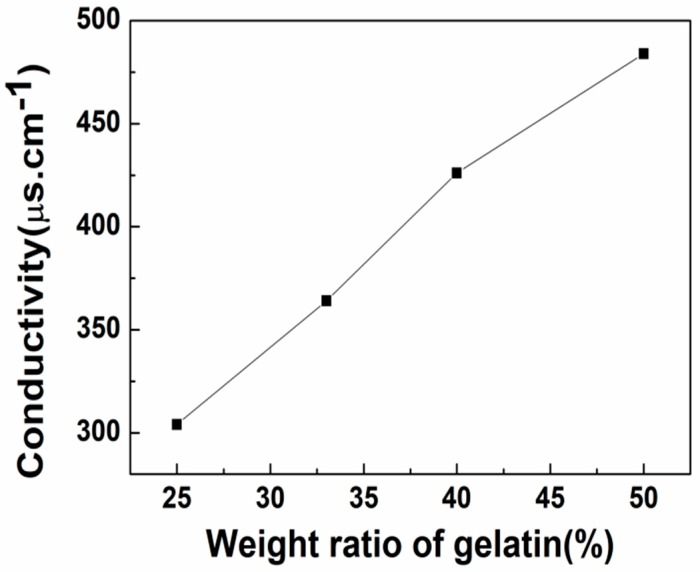
The relationship between the conductivity of blended gelatin/pullulan solutions and the weight ratios of gelatin (total polymer concentration = 25% *w*/*v*).

The effect of the gelatin content on the electrical conductivity of blended gelatin/pullulan solutions was also investigated. The results (Figure 3) showed that the solutions could be more conductive and showed higher values with increased gelatin content. The reason for this could be that gelatin is a polyelectrolytic polymer, and thus the increase of gelatin content could lead to an increase in the conductivity. Moreover, the increase of conductivity can enhance the drag force and promote uniform fiber diameter.

### 3.2. Characterization of Blended Gelatin/Pullulan Nanofiber Membranes

#### 3.2.1. Morphological Characterization of Nanofibers

Gelatin can be dissolved in warm water; however, the solution will become a gel due to the strong H-bonding in room temperature [9]. Therefore, it is impossible for a pure gelatin aqueous solution to be electrospun at room temperature, and the gelatin content should be within a certain range when blended with other polymers. Pullulan is a linear polysaccharide. Incorporation of pullulan was expected to improve the spinnability of an aqueous solution of gelatin. In this study, when the weight ratio of gelatin/pullulan was more than 50/50, electrospinning of the composite aqueous solution failed. The operating parameters of the electrospinning apparatus, including the applied voltage, distance from electrode tip to collector (TCD), and mass flow rate, have certain effects on the morphology of electrospun nanofibers. Varying the TCD will directly affect the flight time of the jet and the electric field strength. The distance should be long enough that the solvent can evaporate thoroughly during the flight time; however, the electric field strength will also decrease at the same time when the distance increases. Moreover, the increase of TCD will lead to the larger diameter of the as-spun fibers [31]. The properties of the solvent, such as volatility and the dielectric effect, will also influence the as-spun fibers. When the flow rate of solvent was too low for the solvent to evaporate sufficiently, the as-spun fibers would tend to merge together and form a film on the collector. Meanwhile, the bending instability of the electrospinning jet was influenced by the dielectric effect. Generally, a stronger dielectric effect would facilitate the reduction of the fiber diameter due to the increased jet path [32].

**Figure 4:**
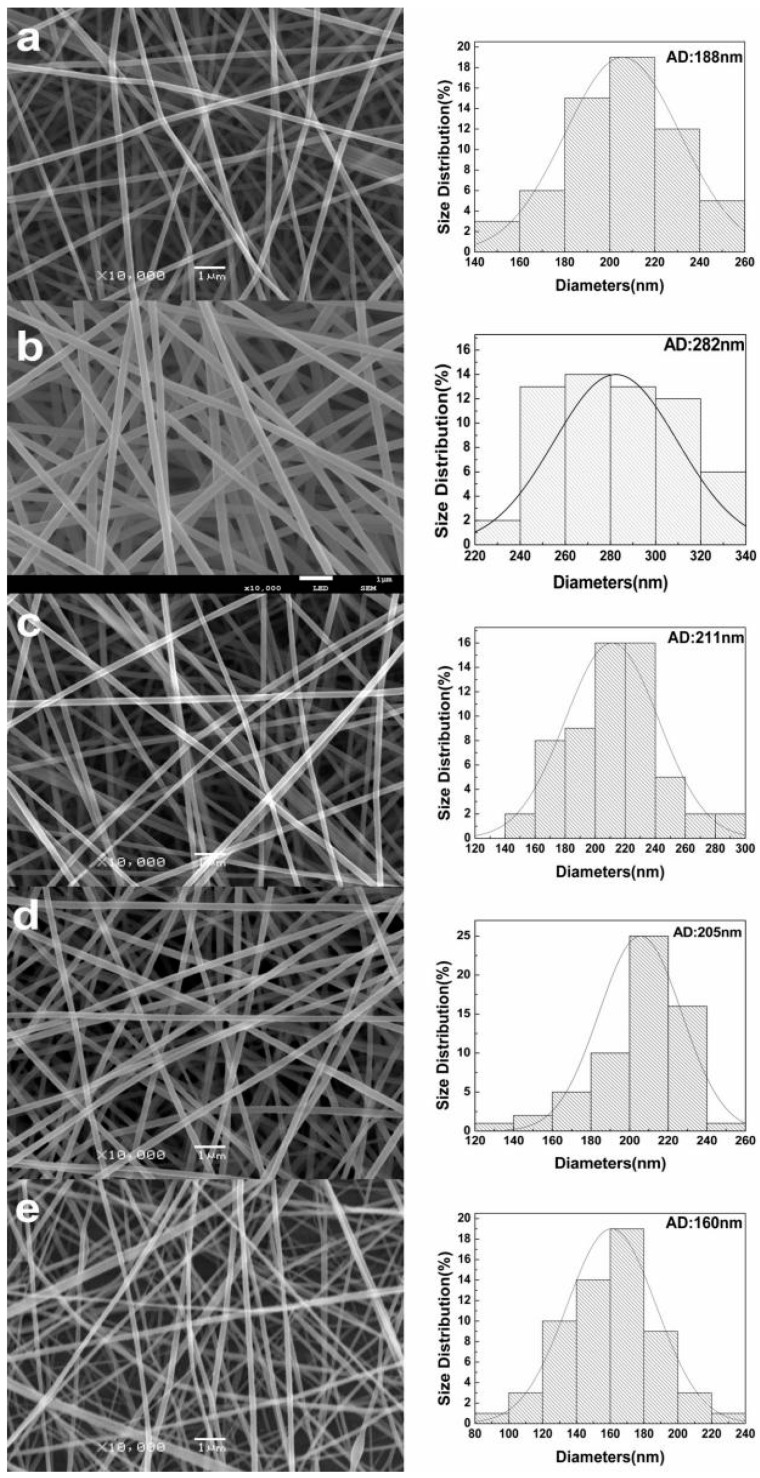
Images and diameter distribution of gelatin/pullulan nanofibers with different concentrations and mass ratios: (**a**) concentration = 20%; mass ratio = 25/75; (**b**) 25%, 25/75; (**c**) 25%, 33/67; (**d**) 25%, 40/60; (**e**) 25%, 50/50.

**Figure 5:**
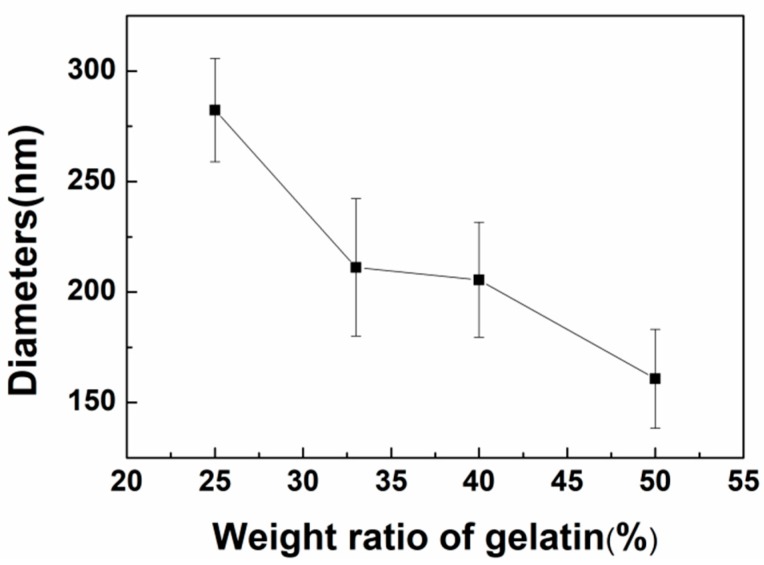
The relationship between the average diameters of gelatin/pullulan nanofibers and weight ratios of gelatin (total polymer concentration = 25% *w*/*v*).

In this work, the applied voltage and TCD were fixed at 20 kV and 20 cm, respectively, during the whole experiment. SEM images, as shown in Figure 4a,b, demonstrate the effect of polymer concentrations on the diameter of nanofibers. The diameter and diameter distribution of the fibers were quantitatively analyzed from the respective microscopic images. It was found that the solution concentration changed from 20% to 25% at the same mass ratio, and the average fiber diameter increased from 188 to 282 nm. As shown in Figure 4, the obtained fibers were bead-free and uniform at the concentrations of 20% and 25% *w*/*v*. Meanwhile, it can be seen that the average diameters of the as-spun fibers showed a decreasing trend with the increase of gelatin content, as shown in Figure 5. The addition of polyelectrolytes increases the charge density of the ejected jets, which leads to a stronger elongation force, and thus the obtained fibers would be thinner and more uniform [33]. Meanwhile, the diameter distribution was narrowed with the increase of conductivity.

#### 3.2.2. FTIR Analysis of Gelatin/Pullulan Nanofibers

**Figure 6:**
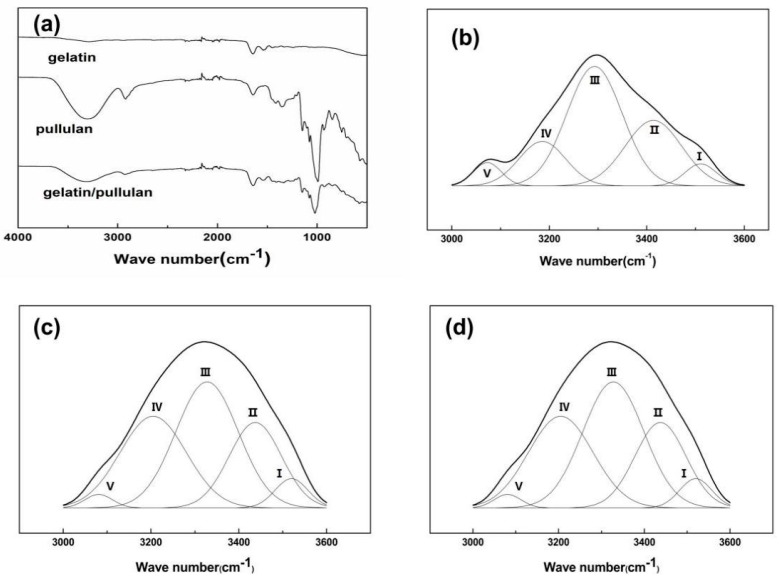
Spectra of pullulan, gelatin, and gelatin/pullulan blend (**a**), and Gauss curve fitting by hydrogen bonds of composite materials: (**b**) gelatin; (**c**) pullulan, and (**d**) gelatin/pullulan blend.

The FTIR spectra of gelatin, pullulan, and gelatin/pullulan blends were recorded as shown in Figure 6a. The typical absorption peak of gelatin occurred at 3277 cm^−1^, which represented the absorption peak of the N–H stretching vibration and the hydroxyl group (O–H) of gelatin. The characteristic peak caused by the C–N stretching vibration of gelatin was found at 1240 cm^−1^, which was relatively weak. The characteristic of the α-configuration of α-d-glucopyranose units occurred at 847 cm^−1^. The band seen at 3330 cm^−1^ represents the hydroxyl group (O–H) of pullulan. The absorption peak of the hydroxyl group of blended gelatin/pullulan was shifted to the lower position of 3316 cm^−1^. This indicated that hydrogen bonds were created between gelatin and pullulan (i.e., hydrogen bonds were formed between the N–H and O–H of the gelatin molecules and the O–H of pullulan molecules).

In order to explore the variation of hydrogen bonds, the spectra of 3000–3600 cm^−1^ were smoothed by the Savitzky–Golay method in Origin 8.5 (Origin Lab, Northampton, Massachusetts, USA). As shown in Figure 6b–d and Table 1, the positions, strength, and subpeak distributions of different types of hydrogen bonds were fitted by the Gaussian method in the range of 3000–3600 cm^−1^. The proportion of intermolecular hydrogen bonds increased, while the ratio of intramolecular hydrogen bonds decreased. The data reported in this paper shows that the interaction between gelatin and pullulan composites was mainly via intermolecular hydrogen bonds, which were developed by breaking intramolecular hydrogen bonds.

#### 3.2.3. Analysis of Tensile Strength of Nanofibers

**Figure 7:**
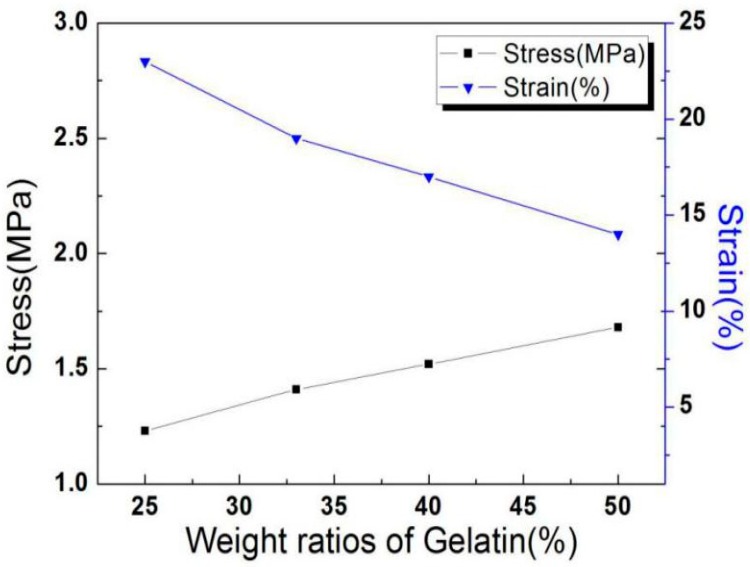
The relationship between the stress and strain of electrospun gelatin/pullulan composite membranes and the weight ratios of gelatin (total polymer concentration = 25% *w*/*v*).

The tensile strength of electrospun gelatin/pullulan composite nanofibrous membranes in terms of gelatin content is shown in Figure 7. The tensile strength increased proportionally with the increasing gelatin content. The reason for this could be the increase of interfacial adhesion between gelatin and pullulan [23]. The results demonstrated that the blend ratio had a significant influence on the mechanical properties of the composite nanofibers. However, the elongation at break of the electrospun gelatin/pullulan composite nanofibrous membranes gradually decreased with the increase of gelatin content, which was caused by strong interactions such as hydrogen bonds.

## 4. Conclusions

An aqueous solution of gelatin and pullulan was successfully electrospun at room temperature to produce a novel protein–polysaccharide composite nanofibrous membrane with potential to mimic the ECM. The surface tension, viscosity, and conductivity increased as gelatin content increased. SEM analyses showed the fiber diameters mainly depended upon the concentration of the solution. Moreover, the conductivity of the solution with the same concentration plays a more important role in influencing the diameters of the as-spun fibers. It was found that average diameter increased when the solution concentration changed from 20% to 25%. Meanwhile, the average diameter had a decreasing trend with the increase of gelatin content due to the characteristics of the polyelectrolyte. FTIR analyses showed the coexistence of gelatin and pullulan molecules due to hydrogen bonding, and then analyzed the fitting results of various kinds of hydrogen bonds. It can be seen that the ratio of intermolecular hydrogen bonds increased while the ratio of intramolecular hydrogen bonds decreased after gelatin was mixed with pullulan. The tensile strength increased proportionally with the increase of gelatin content, while the elongation at break showed the opposite trend. The electrospun gelatin/pullulan composite nanofibers would be a good candidate in the application of tissue engineering.

## Figures and Tables

**Table 1:** The fitting results of various kinds of hydrogen bonds.

Sample	Hydrogen Bond Type	Abbreviations	Wave Number (cm^−1^)	Relative Strength (%)	
Gelatin	Intramolecular hydrogen bond	(II) OH. . .OH	3413	26.98	42.12
		(IV) Annular polymer	3185	15.14	
	Intermolecular hydrogen bond	(III) OH. . .ether O	3292	48.10	57.88
		(I) OH. . .π	3510	5.04	
		(V) OH. . .N	3073	4.74	
Pullulan	Intramolecular hydrogen bond	(II) OH. . .OH	3444	18.32	46.88
		(IV) Annular polymer	3194	28.56	
	Intermolecular hydrogen bond	(III) OH. . .ether O	3325	46.01	53.12
		(I) OH. . .π	3522	4.36	
		(V) OH. . .N	3094	2.75	
Gelatin/ pullulan	Intramoleculr hydrogen bond	(II) OH. . .OH	3435	20.84	41.34
		(IV) Annular polymer	3193	20.50	
	Intermolecular hydrogen bond	(III) OH. . .ether O	3312	49.23	58.66
		(I) OH. . .π	3504	0.78	
		(V) OH. . .N	3084	8.65	
